# A new fruit fly optimization algorithm enhanced support vector machine for diagnosis of breast cancer based on high-level features

**DOI:** 10.1186/s12859-019-2771-z

**Published:** 2019-06-10

**Authors:** Hui Huang, Xi’an Feng, Suying Zhou, Jionghui Jiang, Huiling Chen, Yuping Li, Chengye Li

**Affiliations:** 1 School of Marine Science and Technology, Northwestern Polytechnical University, Xi’an, 710072 China; 2 Pathology Department of Wenzhou People’s Hospital, Wenzhou, 325035 China; 3 Zhijiang College of Zhejiang University of Technology, Hangzhou, 310024 China; 4 Department of Computer Science, Wenzhou University, Wenzhou, 325035 China; 5 Department of Pulmonary and Critical Care Medicine, The First Affiliated Hospital of Wenzhou Medical University, Wenzhou, 325000 China

**Keywords:** Support vector machine, Parameter optimization, Fruit fly optimization, Levy flight, Breast cancer diagnosis

## Abstract

**Background:**

It is of great clinical significance to develop an accurate computer aided system to accurately diagnose the breast cancer. In this study, an enhanced machine learning framework is established to diagnose the breast cancer. The core of this framework is to adopt fruit fly optimization algorithm (FOA) enhanced by Levy flight (LF) strategy (LFOA) to optimize two key parameters of support vector machine (SVM) and build LFOA-based SVM (LFOA-SVM) for diagnosing the breast cancer. The high-level features abstracted from the volunteers are utilized to diagnose the breast cancer for the first time.

**Results:**

In order to verify the effectiveness of the proposed method, 10-fold cross-validation method is used to make comparison among the proposed method, FOA-SVM (model based on original FOA), PSO-SVM (model based on original particle swarm optimization), GA-SVM (model based on genetic algorithm), random forest, back propagation neural network and SVM. The main novelty of LFOA-SVM lies in the combination of FOA with LF strategy that enhances the quality for FOA, thus improving the convergence rate of the FOA optimization process as well as the probability of escaping from local optimal solution.

**Conclusions:**

The experimental results demonstrate that the proposed LFOA-SVM method can beat other counterparts in terms of various performance metrics. It can very well distinguish malignant breast cancer from benign ones and assist the doctor with clinical diagnosis.

## Background

Breast cancer is the most common cancer and the leading cause of cancer death among females [1]. Early detection and diagnosis is the key to controlling the disease and to improving the survival rate, and pathological diagnosis is the most reliable gold standard of all kinds of methods. Traditional diagnostic methods mostly rely on clinicians’ personal experience and the diagnostic results may be subjectivism with certain probability. In recent years, computational diagnostic tools and artificial intelligence techniques provide automated procedures for objective judgments by making use of quantitative measures and machine learning techniques for medical diagnosis [2–11]. Similarly, the methods based on artificial intelligence technology for diagnosis of breast cancer have been proposed. Maglogiannis et al. [12] presented using support vector machine (SVM) for diagnosing the breast cancer both on Wisconsin Diagnostic Breast Cancer and the Wisconsin Prognostic Breast Cancer datasets. Kaya et al. [13] proposed a novel approach based on rough set and extreme learning machine for distinguishing the benign or malignant breast cancer. Akay et al. [14] proposed a novel SVM combined with feature selection for breast cancer diagnosis. The experimental results indicate that the proposed method can perform well in terms of accuracy, sensitivity and specificity. Given recent advances on digitized histological studies, it is now possible to use histological tissue patterns with artificial intelligence techniques-aided image analysis to facilitate disease classification [15]. In general, accurate pathological diagnosis of breast cancer depends on features, which are extracted from histopathology images. There are a lot of works for diagnosis of breast cancer based on histopathology images’ features.

Kuse et al. [16] extracted texture features from the cells to train a SVM classier that is used to classify lymphocytes and non-lymphocytes. Dundar et al. [17] proposed to segment cell regions by clustering the pixel data and to identify individual cells by a watershed-based segmentation algorithm, and a proposed MIL approach was used to identify the stage of breast lesion. Sparks et al. [18] presented a CBIR system that leveraged a novel set of explicit shape features which accurately described the similarity between the morphology of objects of interest. Basavanhally et al. [19] presented a novel framework that classifies entire images based on quantitative features extracted from fields of view of varying sizes. In each FOV, cancer nuclei were automatically detected and used to construct graphs (Voronoi Diagram, Delaunay Triangulation, Minimum Spanning Tree). Features describing spatial arrangement of the nuclei were extracted and used to train a boosted classifier that predicts image class for each FOV size.

In all aforementioned works, an objective phenomenon can be found that these studies were usually conducted on the low-level features on image pixels and the high-level ones were discard, which means that these studies may not express prior medical knowledge. Therefore, in this paper, we proposed to diagnose the breast cancer using the high-level features which were defined based on the prior medical knowledge. This definition relies on two very experienced pathologists. Because these features include the experience of doctors, doctors with clinical experience have a high ability to differentiate between breast tumors and breast cancer in general, and have better comprehensibility. We extracted a set of high-level features, including 13 key features, which were the basis for the classification and grading of breast pathology. Based on these features, the pathological data of 470 cases were analyzed by two pathological experts. Then, we proposed a novel learning framework based on SVM for distinguishing malignant breast cancer from the healthy ones. As we all know, the two key parameters in classic SVM are penalty factor and width of kernel function, which traditionally treated by means of grid search and gradient descent. However, these methods are easy to get into local optimal solutions. Recently, some bio-inspired metaheuristic search algorithms (such as genetic algorithms (GA) [20–23], particle swarm optimization algorithms (PSO) [24–27], the fruit fly optimization (FOA) [28], moth-flame optimization (MFO) [29]) have made it easier to find the global optimal solution. As a new member of the swarm-intelligence algorithms, FOA [30] is inspired by the foraging behavior of real fruit flies. The FOA has certain outstanding merits, such as a simple computational process, simple implementation, and easy understanding with only a few parameters for tuning. Due to its good properties, FOA has become a useful tool for many real-world problems [10, 28, 31–33].

Compared with gradient descent method and grid search method, like other swarm intelligence methods [34, 35], FOA is a global optimization method, which can find the global optimal solution or approximate optimal solution more easily. However, the traditional FOA algorithm has the possibility of falling into the local optimal solution for complex optimization problems, and the convergence rate is not very ideal. Therefore, this paper introduces the Levy flight (LF) strategy to update the positions of fruit flies to further improve its convergence speed, while reducing the probability of FOA falling into the local optimal. LF strategy has been used widely to enhance the lots of metaheuristic algorithms [36–41]. The principle of LF strategy can ensure the diversity of algorithms in the process of optimization [42–44] and improve the convergence rate. In this study, the improved FOA method, LFOA, was utilized to optimize the two key parameters pair including penalty factor and width of kernel function in SVM method and obtain the optimal model (LFOA-SVM). Furthermore, this model will be investigated to diagnose the breast cancer on high-level features dataset. As far as we know, this paper is the first to solve the parameter optimization problem of SVM with LFOA. In the experiment, a 10-fold cross-validation method was used on data to make detailed comparison between LFOA--SVM, FOA-SVM (model based on the primitive fruit fly optimization model), GA-SVM (model based on genetic algorithms), PSO-SVM (model based on particle swarm optimization algorithms), random forest (RF), back propagation neural network (BPNN) and SVM. The experimental results demonstrated that the proposed LFOA-SVM was superior to other methods in terms of classification accuracy, Mathews correlation coefficient (MCC), sensitivity and specificity.

The rest of this paper is organized as follows. In Preliminaries Section background information used in the study was introduced. In Methods Section the detailed implementation of the proposed method was presented. In Results and discussion Section, experimental designs, results and discussion were delivered. Finally, in Conclusion Section the conclusions and recommendations for future work were summarized.

## Preliminaries

### Support vector machine

Support Vector Machine (SVM) [45] is a supervised learning model and related learning algorithm for analyzing data in classification and regression analysis. Given a set of training instances, each training instance is marked as one or the other of two classes, the SVM training algorithm creates a model that assigns a new instance to one of two classes, making it a non-probabilistic binary linear classifier.

The SVM model is to represent instances as points in space, so that the mapping allows instances of separate categories to be separated by as wide and distinct intervals as possible. Then, new instances are mapped to the same space and the category is predicted based on which side they fall in the interval. In addition to linear classification, SVM can also use the so-called kernel technique to effectively perform nonlinear classification, mapping its input implicitly into the high-dimensional feature space.

More formally, support vector machines construct hyperplanes in high-dimensional or infinite-dimensional spaces. Which can be used for classification, regression or other tasks. Intuitively, the farther away the nearest training data point is, the better, because this can reduce the generalization error of the classifier.

### Fruit-Fly optimization algorithms

The fruit fly optimization algorithm (FOA) [30] was a meta-heuristic algorithm which is inspired by the foraging behavior of fruit fly. Fruit fly relies on vision and smell to position food during foraging. FOA searches for solution space by mimicking the way of fruit fly flight when solving optimization problems. In FOA, first, the fruit fly population (candidate solution) is randomly generated in the solution space, and then each fruit fly will update its position according to the flight mode of the fruit fly. Fruit fly population continuously improves the fitness of the population (quality of solution) during the iterative process.

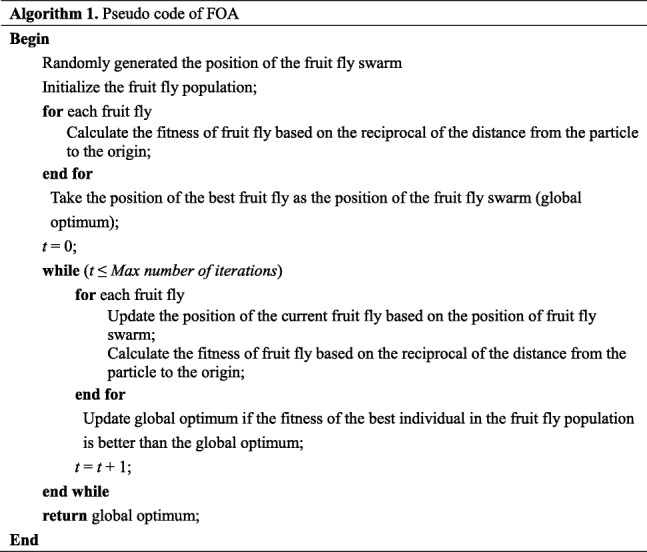



### Levy flight

Levy flight (LF) mechanism is often used to improve meta-heuristics because its characteristics are similar to the movement of many animals in nature. The phenomena is called Levy statistics [46]. The LF is essentially stochastic non-Gaussian walks. Its step value is dispersed relative to Levy stable distribution. Levy distribution can be represented as the following equation:

1 $$ Levy(s)\sim {\left|s\right|}^{-1-\beta },0<\beta \le 2 $$

*β* represents an important Levy index to adjust the stability, *s* is the step length.

## Methods

### Levy flight enhanced FOA (LFOA)

Levy’s flight is characterized by short steps and random directions. This feature can effectively avoid the whole population falling into local optimum, thus enhancing the global detection ability of the algorithm. In this paper, we have introduced the LF strategy into to FOA to explore the search space more efficiently. The new position is updated according to the following rule.

2 $$ {X}_i^{levy}={X}_i+{X}_i\oplus levy(s) $$

where ${X}_i^{levy}$ is the new position of the *i*th search agent *X*_*i*_ after updating.

### Proposed LFOA-SVM model

**Fig. 1:**
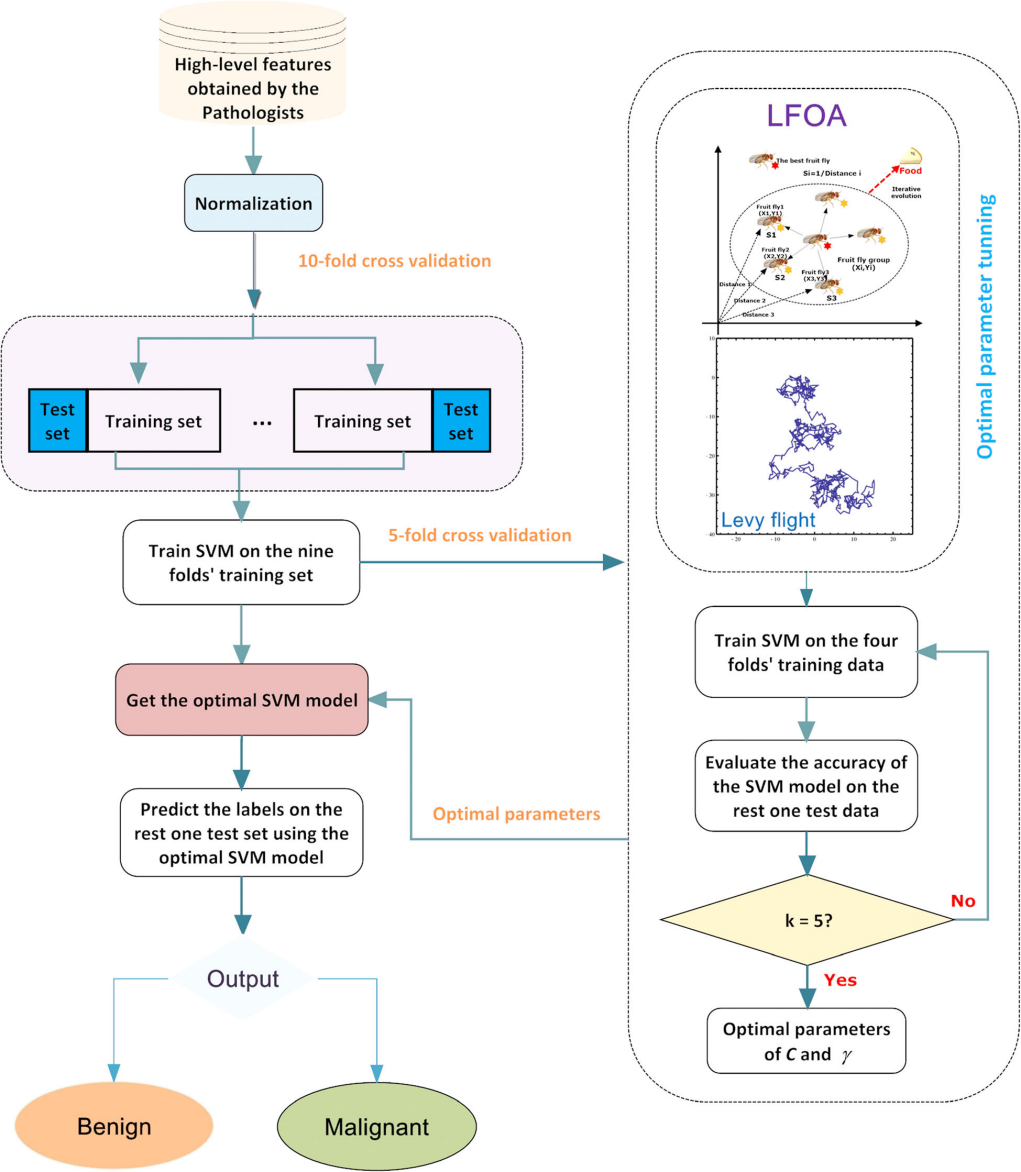
Flowchart of LFOA-SVM

This study proposes a novel evolutionary SVM that employs the LFOA strategy, and the resultant LFOA-SVM model can adaptively determine the two key hyper-parameters for SVM. The general framework of the proposed method is demonstrated in Fig. 1. The proposed model is primarily comprised of two procedures: the inner parameter optimization and the outer classification performance evaluation. During the inner parameter optimization procedure, the SVM parameters are dynamically adjusted by the LFOA technique via the 5-fold cross validation (CV) analysis. Then, the obtained optimal parameters are fed to the SVM prediction model to perform the classification task for breast cancer diagnosis in the outer loop using the 10-fold CV analysis. The classification accuracy was used as the fitness function.

3 $$ fitness=\left({\sum}_{i=1}^K AC{C}_i\right)/k $$

where *ACC*_*i*_ represents the average accuracy achieved by the SVM classifier via 5-fold CV.

The main steps conducted by the LFOA-SVM are described in detail as follows:

- Step 1: Initialize the input parameters for LFOA, include population size, maximum number of iterations, upper bound of the variables, and lower bound of the variables, the dimension of the problem.
- Step 2: Randomly generated the position of the fruit fly swarm based on the upper and lower bounds of the variables.
- Step 3: Generate initial population for LFOA based on the position of the fruit fly swarm.
- Step 4: Evaluate the fitness of all fruit flies in population by SVM with the position of fruit fly as parameters.
- Step 5: Take the position of the best fruit fly as the position of the fruit fly swarm (global optimum).
- Step 6: Update the position of each fruit fly in the swarm with Levy-flight mechanism and evaluate the fitness of the fruit fly.
- Step 7: Update global optimum if the fitness of the best individual in the fruit fly population is better than the global optimum.
- Step 8: Update iteration *t*, *t* = *t* + 1. If *t* larger than maximum number of iterations, go to step 6.
- Step 9: Return the global optimum as the optimal SVM parameter pair (*C*, *γ*).

## Results and discussion

### Data description

The data were collected from Wenzhou people’s Hospital from 2004 to 2015. Four hundred seventy objects have been selected as the research objects. There are 232 benign cases and 238 malignant cases. Based on the prior medical knowledge of the classification and grading of breast pathology, we proposed a set of features descriptor with the help of two well-experienced pathologist from Wenzhou people’s hospital of China. A total of 14 key features were included and quantified in this study. Table 1 gives the brief description and quantization of these features.

**Table 1:** The brief descriptions and quantization of features used in this study

Feature	Brief description	Quantization
X1	Age	<=40: 1; > 40: 2
X2	Locality	unilateral: 1; bilateral: 2
X3	Boundary	clear: 1; not clear: 2; obscure: 3
X4	Calcification	no: 1; yes: 2
X5	Cystic	no: 1; yes: 2
X6	Nucleus/cytoplasm ratio(N/C)	low: 1; medium: 2; high: 3
X7	Nucleolus size	small: 1; big: 2
X8	Nucleolus number	Single: 1; Multi: 2
X9	Cell density	low: 1; medium: 2; high: 3
X10	Presence of fiber focal	no: 1:; yes: 2
X11	Inflammatory cell infiltration	few: 1; more: 2; many: 3
X12	Infiltration of the gland	no: 1; yes: 2
X13	Perineural invasion	no: 1; yes: 2
X14	Glands clearance around	no: 1; yes: 2

### Experimental setup

The LFOA-SVM, FOA-SVM, PSO-SVM, GA-SVM, RF, BPNN and ELM classification models were implemented using the MATLAB platform. For SVM, the LIBSVM implementation was utilized, which was originally developed by Chang and Lin [47]. For RF, the code package from https://code.google.com/archive/p/randomforest-matlab/ was adopted. We implemented the LFOA, FOA, GA and PSO from scratch. The computational analysis was conducted on a Windows Server 2008 operating system with Intel Xeon CPU E5–2650 v3(2.30 GHz) and 16GB of RAM.

In order to conduct an accurate comparison, the same number of generations and the same population swarm size were used for FOA, PSO, and GA. According to the preliminary experiment, when the number of generations and the swarm size are set to 250 and 8, respectively, the involved methods produce a satisfactory classification performance. For the metaheuristic methods, the same searching range of the parameters *C∈*[2^− 5^, 2^15^] and *γ∈* [2^–15^, 2] was used. The parameter settings for relevant algorithms are shown in Table 2.

**Table 2:** The parameter settings for the relevant methods

Algorithm	Parameter	Value
LFOA	*ax, ay*	20,20
	*bx, by*	10,10
FOA	*ax, ay*	20,20
	*bx, by*	10,10
GA	*crossover*	0.4
	*mutation*	0.01
PSO	*c*_1_ *, c*_2_	2
	*w*	1
	*v*_*max*_	6
MFO	*a*	*a*∈[−1–2]
	*b*	1
BA (bat algorithm)	*Q*	*Q*∈[0 2]
	*A*	0.5
	*r*	0.5
DA (dragon fly algorithm)	*w*	*w*∈[0.9 0.2]
	*s*	0.1
	*a*	0.1
	*c*	0.7
	*f*	1
	*e*	1
FPA (flower pollination algorithm)	*p*	0.8
	*λ*	1.5
SCA (sine cosine algorithm)	*a*	2
RF	*Number of trees (ntree)*	500
	*Number of variables (mtry)*	3
BPNN	*Number of hidden neurons*	8
	*Type of activation function*	Sigmoid
	*Learning algorithm*	tranlim
ELM	*Number of hidden neurons*	50
	*Type of activation function*	Sigmoid

The *k*-fold CV [48] was used to evaluate the classification performance of the model. A nested stratified 10-fold CV was used for the purposes of this study [49]. To evaluate the proposed method, commonly used evaluation criteria such as classification accuracy (ACC), sensitivity, specificity and Matthews Correlation Coefficients (MCC) were analyzed.

### Benchmark function verification

To verify the performance of the proposed method LFOA, we use a common set of 23 benchmark functions, including unimodal, multimodal, and fixed-dimension multimodal. The formulas and brief descriptions of these functions can be seen in Tables 3, 4 and 5.

**Table 3:** Unimodal benchmark functions

Function	Dim	Range	*f*_min_
${f}_1(x)={\sum}_{i=1}^n{x_i}^2$	30	[− 100, 100]	0
${f}_2(x)={\sum}_{i=1}^n\mid {x}_i\mid +{\prod}_{i=1}^n\mid {x}_i\mid$	30	[− 10, 10]	0
${f}_3(x)={\sum}_{i=1}^n{\left({\sum}_{j-1}^i{x}_j\right)}^2$	30	[− 100, 100]	0
*f*_4_(*x*) = max_*i*_{| *x*_*i*_| , 1 ≤ *i* ≤ *n*}	30	[− 100, 100]	0
${f}_5(x)={\sum}_{i=1}^{n-1}\left[100{\left({x}_{i+1}-{x_i}^2\right)}^2+{\left({x}_i-1\right)}^2\right]$	30	[− 30, 30]	0
${f}_6(x)={\sum}_{i=1}^n{\left(\left[{x}_i+0.5\right]\right)}^2$	30	[− 100, 100]	0
${f}_7(x)={\sum}_{i=1}^n{ix_i}^4+ random\left[0,1\right)$	30	[− 1.28, 1.28]	0

**Table 4:** Multimodal benchmark functions

Function	Dim	Range	*f*_min_
${f}_8(x)={\sum}_{i=1}^n-{x}_i\sin \left(\sqrt{\mid {x}_i\mid}\right)$	30	[− 500,500]	−418.9829 × 5
${f}_9(x)={\sum}_{i=1}^n\left[{x_i}^2-10\cos \left(2\pi {x}_i\right)+10\right]$	30	[− 5.12,5.12]	0
${f}_{10}(x)=-20\exp \left(-0.2\sqrt{\frac{1}{n}{\sum}_{i=1}^n{x_i}^2}\right)-\exp \left(\frac{1}{n}{\sum}_{i=1}^n\cos \left(2\pi {x}_i\right)\right)+20+e\kern0.50em$	30	[− 32,32]	0
${f}_{11}(x)=\frac{1}{4000}{\sum}_{i=1}^n{x_i}^2-{\prod}_{i=1}^n\cos \left(\frac{x_i}{\sqrt{i}}\right)+1$	30	[− 600,600]	0
$$ {\displaystyle \begin{array}{l}{f}_{12}(x)=\frac{\pi }{n}\left\{10\sin \left(\pi {y}_1\right)+{\sum}_{i=1}^{n-1}{\left({y}_i-1\right)}^2\left[1+10{\sin}^2\left(\pi {y}_{i+1}\right)\right]+{\left({y}_n-1\right)}^2\right\}+{\sum}_{i=1}^nu\left({x}_i,10,100,4\right)\\ {}{y}_i=1+\frac{x_i+1}{4}\\ {}u\left({x}_i,a,k,m\right)\left\{\begin{array}{l}k{\left({x}_i-a\right)}^m\kern0.75em {x}_i>a\\ {}0\kern3.75em -a<{x}_i<a\\ {}k{\left(-{x}_i-a\right)}^m\kern0.75em {x}_i<-a\end{array}\right.\\ {}\end{array}} $$	30	[− 50,50]	0
$$ {\displaystyle \begin{array}{l}{f}_{13}(x)=0.1\left\{{\sin}^2\left(3\uppi {x}_1\right)+{\sum}_{\mathrm{i}=1}^{\mathrm{n}}\left({x}_{\mathrm{i}}\hbox{-} 1\right]\Big){}^2\left[1+{\sin}^2\left(3\uppi {x}_{\mathrm{i}}+1\right)\right]+{\left({x}_{\mathrm{n}}-1\right)}^2\left[1+{\sin}^2\left(2\uppi {x}_{\mathrm{n}}\right)\right]\right\}\\ {}+{\sum}_{\mathrm{i}=1}^{\mathrm{n}}u\left({x}_{\mathrm{i}},5,100,4\right)\end{array}} $$	30	[− 50,50]	0

**Table 5:** Fixed-dimension multimodal benchmark functions

Function	Dim	Range	*f*_min_
${f}_{14}(x)={\left(\frac{1}{500}+{\sum}_{j=1}^{25}\frac{1}{j+{\sum}_{i=1}^2{\left({x}_i-{a}_{ij}\right)}^6}\right)}^{-1}$	2	[− 65,65]	1
${f}_{15}(x)={\sum}_{i=1}^{11}{\left[{a}_i-\frac{x_1\left({b}_i^2+{b}_i{x}_2\right)}{b_i^2+{b}_i{x}_3+{x}_4}\right]}^2$	4	[− 5, 5]	0.00030
${f}_{16}(x)=4{x}_1^2-2.1{x}_1^4+\frac{1}{3}{x}_1^6+{x}_1{x}_2-4{x}_2^2+4{x}_2^4$	2	[− 5,5]	−1.0316
${f}_{17}(x)={\left({x}_2-\frac{5.1}{4{\pi}^2}{x}_1^2+\frac{5}{\pi }{x}_1-6\right)}^2+10\left(1-\frac{1}{8\pi}\right)\cos {x}_1+10$	2	[− 5,5]	0.398
$$ {\displaystyle \begin{array}{l}{f}_{18}(x)=\left[1+{\left({x}_1+{x}_2+1\right)}^2\left(19-14{x}_1+3{x}_1^2-14{x}_2+6{x}_1{x}_2+3{x}_2^2\right)\right]\\ {}\times \left[30+{\left(2{x}_1-3{x}_2\right)}^2\times \left(18-32{x}_1+12{x}_1^2+48{x}_2-36{x}_1{x}_2+27{x}_2^2\right)\right]\end{array}} $$	2	[− 2,2]	3
${f}_{19}(x)=-{\sum}_{i=1}^4{c}_i\exp \left(-{\sum}_{j=1}^3{a}_{ij}{\left({x}_j-{p}_{ij}\right)}^2\right)$	3	[1, 3]	−3.86
${f}_{20}(x)=-{\sum}_{i=1}^4{c}_i\exp \left(-{\sum}_{j=1}^6{a}_{ij}{\left({x}_j-{p}_{ij}\right)}^2\right)$	6	[0,1]	−3.32
${f}_{21}(x)=-{\sum}_{i=1}^5{\left[\left(X-{a}_i\right){\left(X-{a}_i\right)}^{\mathrm{T}}+{c}_i\right]}^{-1}$	4	[0,10]	−10.1532
${f}_{22}(x)=-{\sum}_{i=1}^7{\left[\left(X-{a}_i\right){\left(X-{a}_i\right)}^{\mathrm{T}}+{c}_i\right]}^{-1}$	4	[0,10]	−10.4028
${f}_{23}(x)=-{\sum}_{i=1}^{10}{\left[\left(X-{a}_i\right){\left(X-{a}_i\right)}^{\mathrm{T}}+{c}_i\right]}^{-1}$	4	[0,10]	−10.5363

**Fig. 2:**
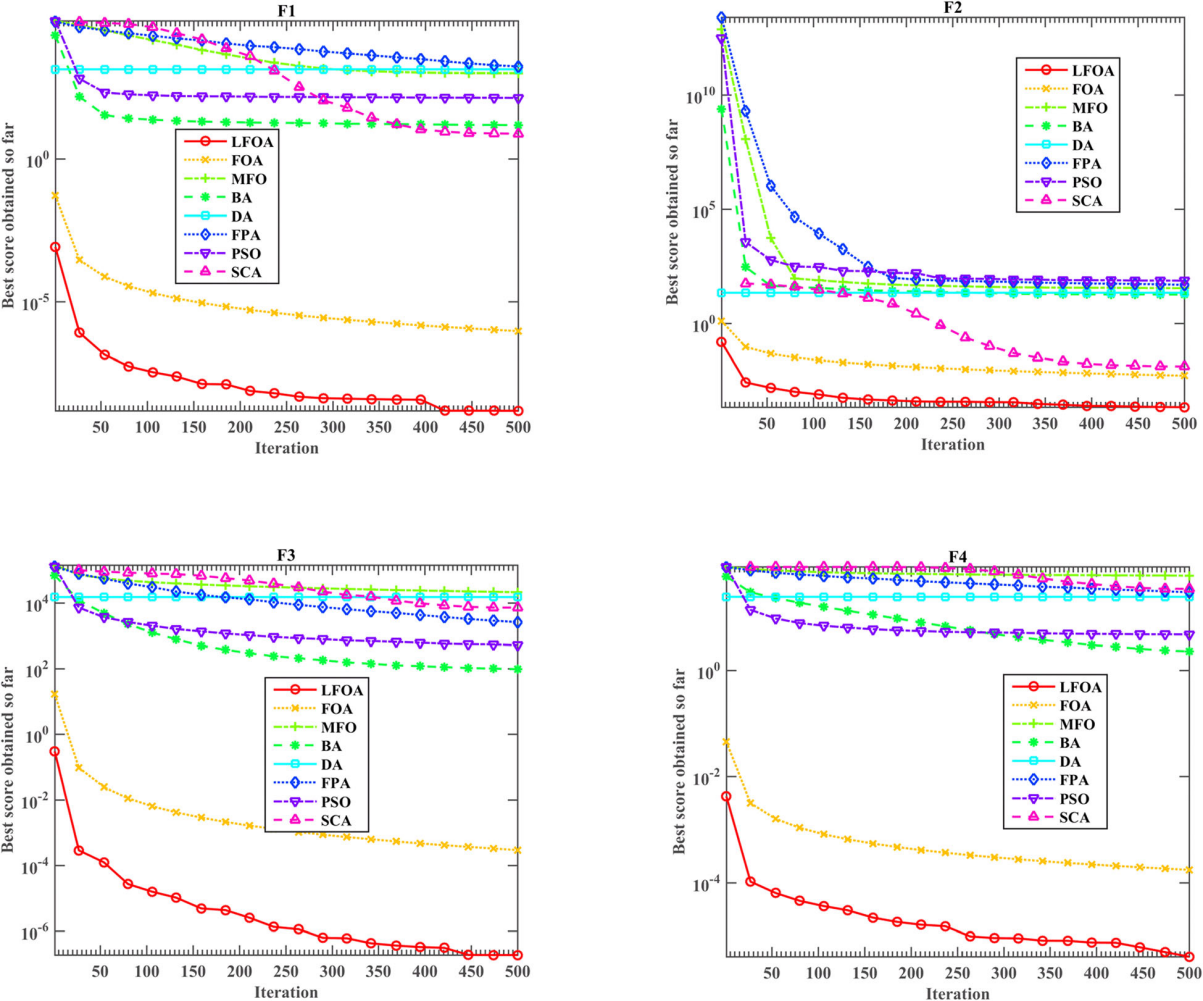
Convergence curves of LFOA and other algorithms for f_1_, f_2_, f_3_ and f_4_

Moreover, the performance of the LFOA is also compared with the original FOA, MFO, BA, DA, FPA, PSO, and SCA. The relevant parameter settings for the algorithm mentioned above for comparison refer to the previous papers, and as shown in Table 2, specific parameter values have been listed. In order to obtain more accurate experimental results, 30 independent experiments are performed on each test function, and the average value is calculated as the final result of each algorithm. The number of iterations and population size of the algorithm are set to 500 and 30, respectively. The results obtained are reported in Table 6 and Fig. 2. The average (Avg.), standard deviation (Std.) and rankings of the different algorithms in solving the *f*_1_-*f*_23_ test functions are displayed in Table 6.

**Table 6:** Results of testing benchmark functions

Function	Metric	LFOA	FOA	MFO	BA	DA	FPA	PSO	SCA
*f*_*1*_	mean	1.53E-09	9.27E-07	1.00E+ 03	1.53E+ 01	1.36E+ 03	1.71E+ 03	1.39E+ 02	7.65E+ 00
	std	3.71E-09	7.92E-09	3.05E+ 03	2.32E+ 00	1.11E+ 03	4.11E+ 02	1.77E+ 01	1.07E+ 01
	rank	1	2	6	4	7	8	5	3
*f*_*2*_	mean	2.20E-04	5.26E-03	3.51E+ 01	1.84E+ 01	2.20E+ 01	4.90E+ 01	7.48E+ 01	1.33E-02
	std	2.25E-04	3.13E-05	2.09E+ 01	1.99E+ 00	1.69E+ 01	1.20E+ 01	1.43E+ 01	1.47E-02
	rank	1	2	6	4	5	7	8	3
*f*_*3*_	mean	1.85E-07	2.91E-04	2.12E+ 04	9.62E+ 01	1.51E+ 04	2.59E+ 03	5.14E+ 02	7.18E+ 03
	std	3.20E-07	2.66E-06	1.25E+ 04	2.99E+ 01	1.13E+ 04	8.39E+ 02	1.13E+ 02	4.60E+ 03
	rank	1	2	8	3	7	5	4	6
*f*_*4*_	mean	4.12E-06	1.76E-04	5.95E+ 01	2.25E+ 00	2.38E+ 01	2.91E+ 01	4.69E+ 00	3.36E+ 01
	std	4.15E-06	7.28E-07	1.27E+ 01	4.90E-01	7.36E+ 00	3.35E+ 00	2.77E-01	1.05E+ 01
	rank	1	2	8	3	5	6	4	7
*f*_*5*_	mean	8.32E+ 00	2.87E+ 01	5.35E+ 06	4.07E+ 03	5.02E+ 05	2.44E+ 05	1.64E+ 05	1.84E+ 05
	std	6.92E+ 00	1.10E-04	2.92E+ 07	1.10E+ 03	7.48E+ 05	9.91E+ 04	3.81E+ 04	8.00E+ 05
	rank	1	2	8	3	7	6	4	5
*f*_*6*_	mean	4.47E-03	7.51E+ 00	1.34E+ 03	1.57E+ 01	1.47E+ 03	1.64E+ 03	1.36E+ 02	2.00E+ 01
	std	3.29E-03	2.67E-05	3.46E+ 03	1.99E+ 00	9.07E+ 02	4.54E+ 02	1.62E+ 01	3.46E+ 01
	rank	1	2	6	3	7	8	5	4
*f*_*7*_	mean	2.41E-04	2.16E-04	2.23E+ 00	1.01E+ 01	4.73E-01	4.75E-01	9.93E+ 01	6.57E-02
	std	2.33E-04	1.16E-04	4.13E+ 00	6.23E+ 00	3.03E-01	1.58E-01	2.44E+ 01	4.57E-02
	rank	2	1	6	7	4	5	8	3
*f*_*8*_	mean	−1.00E+ 03	−9.36E+ 01	−8.83E+ 03	−7.41E+ 03	− 5.19E+ 03	−7.63E+ 03	−6.89E+ 03	−3.78E+ 03
	std	7.16E+ 02	4.62E+ 01	7.31E+ 02	8.71E+ 02	5.72E+ 02	1.67E+ 02	8.35E+ 02	2.90E+ 02
	rank	7	8	1	3	5	2	4	6
*f*_*9*_	mean	3.83E-07	1.84E-04	1.59E+ 02	2.65E+ 02	1.53E+ 02	1.39E+ 02	3.71E+ 02	3.65E+ 01
	mean	7.64E-07	1.86E-06	4.23E+ 01	1.96E+ 01	6.13E+ 01	2.07E+ 01	2.43E+ 01	3.09E+ 01
	rank	1	2	6	7	5	4	8	3
*f*_*10*_	mean	1.65E-05	7.04E-04	1.48E+ 01	4.94E+ 00	9.29E+ 00	1.44E+ 01	8.52E+ 00	1.20E+ 01
	std	2.21E-05	4.06E-06	7.11E+ 00	2.71E+ 00	1.86E+ 00	1.12E+ 00	4.74E-01	9.24E+ 00
	rank	1	2	8	3	5	7	4	6
*f*_*11*_	mean	1.99E-10	6.19E-08	6.89E+ 00	5.98E-01	1.71E+ 01	1.53E+ 01	1.03E+ 00	8.30E-01
	std	5.63E-10	8.26E-10	2.28E+ 01	5.98E-02	1.46E+ 01	3.27E+ 00	5.56E-03	4.20E-01
	rank	1	2	6	3	8	7	5	4
*f*_*12*_	mean	4.13E-04	1.67E+ 00	4.65E+ 00	1.36E+ 01	2.00E+ 03	1.71E+ 02	5.22E+ 00	3.14E+ 02
	std	2.30E-04	5.08E-06	3.79E+ 00	6.00E+ 00	8.44E+ 03	5.53E+ 02	8.48E-01	1.06E+ 03
	rank	1	2	3	5	8	6	4	7
*f*_*13*_	mean	6.07E-03	6.18E-01	1.37E+ 07	2.47E+ 00	1.04E+ 05	9.76E+ 04	2.61E+ 01	4.49E+ 04
	std	3.92E-03	9.19E-02	7.49E+ 07	3.21E-01	2.72E+ 05	1.03E+ 05	4.32E+ 00	1.55E+ 05
	rank	1	2	8	3	7	6	4	5
*f*_*14*_	mean	1.25E+ 01	1.27E+ 01	1.53E+ 00	4.84E+ 00	1.69E+ 00	9.98E-01	2.71E+ 00	1.53E+ 00
	std	9.16E-01	3.27E-15	1.31E+ 00	4.74E+ 00	1.01E+ 00	2.63E-04	2.02E+ 00	8.92E-01
	rank	6	7	2	5	3	1	4	2
*f*_*15*_	mean	4.82E-04	8.04E-04	9.76E-04	5.15E-03	9.75E-03	7.60E-04	1.37E-03	1.01E-03
	std	1.81E-04	2.93E-04	3.11E-04	7.79E-03	1.34E-02	1.05E-04	3.33E-04	3.58E-04
	rank	1	3	4	7	8	2	6	5
*f*_*16*_	mean	−5.77E-01	−1.68E-01	−1.03E+ 00	−1.03E+ 00	−1.03E+ 00	−1.03E+ 00	−1.03E+ 00	−1.03E+ 00
	std	2.82E-01	1.32E-01	6.78E-16	5.25E-04	2.03E-09	1.69E-08	1.82E-03	3.43E-05
	rank	2	3	1	1	1	1	1	1
*f*_*17*_	mean	5.33E-01	1.98E+ 00	3.98E-01	3.98E-01	3.98E-01	3.98E-01	3.99E-01	3.99E-01
	std	2.75E-01	1.01E+ 00	0.00E+ 00	5.57E-04	1.00E-13	6.22E-09	1.13E-03	1.15E-03
	rank	3	4	1	1	1	1	2	2
*f*_*18*_	mean	3.04E+ 01	6.00E+ 02	3.00E+ 00	3.06E+ 00	3.00E+ 00	3.00E+ 00	3.10E+ 00	3.00E+ 00
	std	3.42E+ 00	1.73E-03	1.70E-15	5.86E-02	1.89E-14	7.88E-07	1.29E-01	5.38E-05
	rank	4	5	1	2	1	1	3	1
*f*_*19*_	mean	−3.71E+ 00	−3.63E+ 00	−3.86E+ 00	−3.84E+ 00	−3.85E+ 00	−3.86E+ 00	−3.85E+ 00	−3.85E+ 00
	std	1.20E-01	2.23E-01	2.71E-15	1.50E-02	6.36E-02	1.09E-06	1.37E-02	1.96E-03
	rank	4	5	1	3	2	1	2	2
*f*_*20*_	mean	−2.15E+ 00	−1.81E+ 00	−3.23E+ 00	−2.84E+ 00	− 3.23E+ 00	− 3.32E+ 00	−2.86E+ 00	−2.95E+ 00
	std	5.03E-01	4.24E-01	5.40E-02	1.29E-01	1.22E-01	4.20E-03	1.74E-01	3.53E-01
	rank	6	7	2	5	2	1	4	3
*f*_*21*_	mean	−5.54E+ 00	−3.47E+ 00	−6.55E+ 00	−4.60E+ 00	−9.31E+ 00	−1.01E+ 01	−3.88E+ 00	− 3.29E+ 00
	std	9.25E-01	7.98E-01	3.33E+ 00	2.32E+ 00	1.92E+ 00	4.78E-02	1.14E+ 00	1.82E+ 00
	rank	4	7	3	5	2	1	6	8
*f*_*22*_	mean	−6.16E+ 00	−3.42E+ 00	−7.45E+ 00	−5.51E+ 00	−9.52E+ 00	−1.03E+ 01	−4.25E+ 00	−4.00E+ 00
	std	1.39E+ 00	8.57E-01	3.49E+ 00	2.86E+ 00	2.00E+ 00	1.06E-01	1.26E+ 00	1.68E+ 00
	rank	4	8	3	5	2	1	6	7
*f*_*23*_	mean	−5.55E+ 00	−3.51E+ 00	−8.54E+ 00	−6.10E+ 00	−9.64E+ 00	−1.04E+ 01	−4.02E+ 00	− 4.20E+ 00
	std	8.64E-01	7.50E-01	3.41E+ 00	3.15E+ 00	2.04E+ 00	1.28E-01	1.27E+ 00	1.24E+ 00
	rank	5	8	3	4	2	1	7	6
Sum of rank		59	88	101	89	104	88	108	99
Average rank		2.5652	3.8261	4.3913	3.8696	4.5217	3.8261	4.6957	4.3043
Overall rank		1	2	5	3	6	2	7	4

As shown in Table 6, on the seven unimodal functions, according to the results of the improved LFOA and other algorithms, it can be clearly seen that except for the function *f*_7_, the results achieved on *f*_1_-*f*_6_ is better than the original FOA and the other six algorithms. For *f*_7_, the FOA performs well for 30-dimension problem. For six multimodal functions, the LFOA method surpasses the other competitors on *f*_9_-*f*_13_. From the results for *f*_8_, although our improved algorithm LFOA could not search much better solutions, there is no doubt that LFOA is still very competitive compared to the original FOA. For ten fixed-dimension multimodal functions, LFOA has attained the exact optimal solutions for 30-dimension problem *f*_15_. For other nine functions (*f*_14_ and *f*_16_-*f*_23_), although in dealing with some problems the improved LFOA is not better than other methods, it is observed that the optimization effect of proposed LFOA is still improved compared with the original FOA. Moreover, based on rankings, the LFOA is the best overall technique and the overall ranks show that FOA, FPA, BA, SCA, MFO, DA, PSO algorithms are in the next places, respectively.

**Fig. 3:**
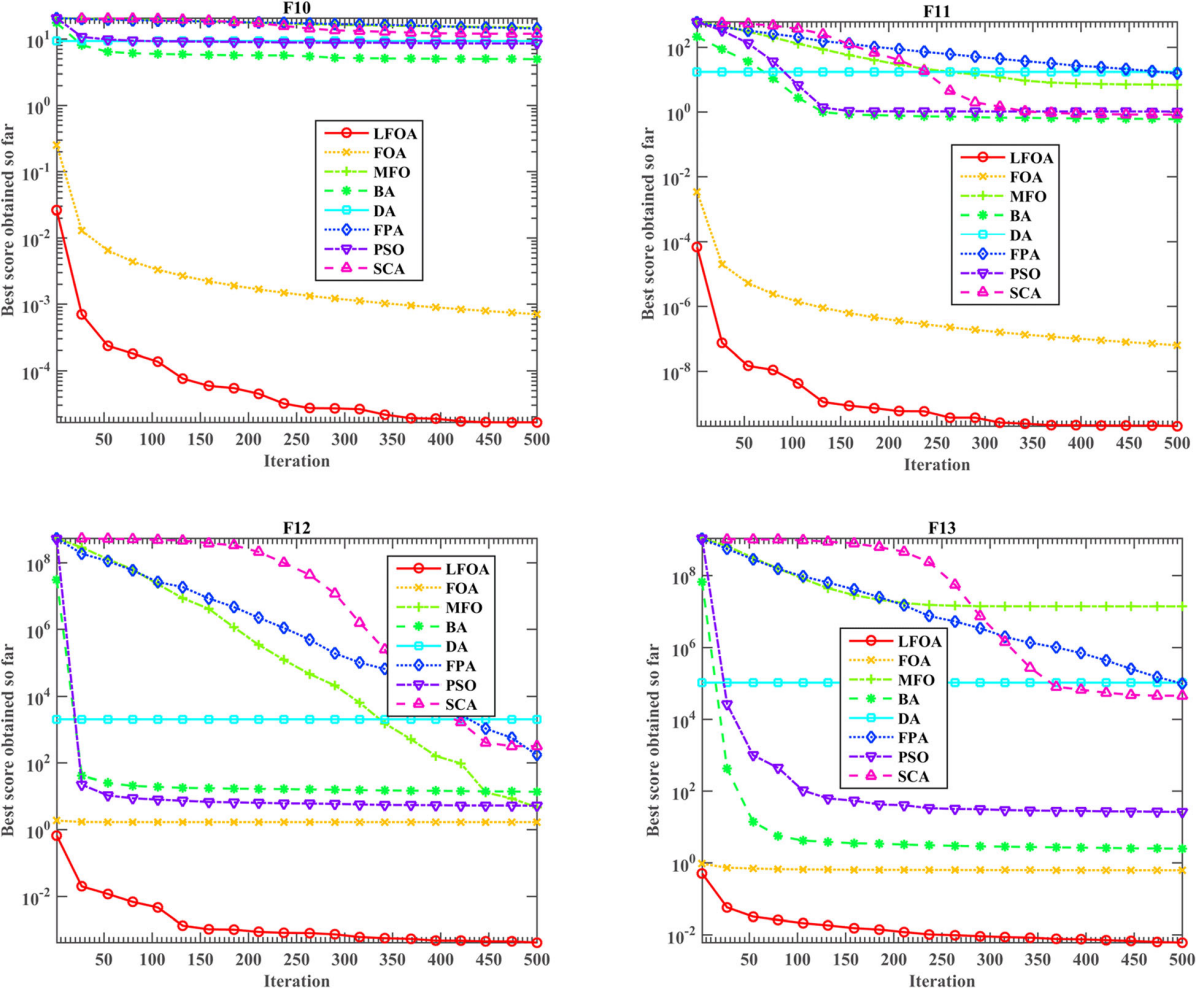
Convergence curves of LFOA and other algorithms for f_10_, f_11_, f_12_ and f_13_

The convergence trends of LFOA and other methods for different test functions (*f*_1_, *f*_2_, *f*_3_, *f*_4_, *f*_10_, *f*_11_, *f*_12_ and *f*_13_) are depicted in Figs. 2 and 3

. From *f*_1_, it can be clearly seen that LFOA can take the lead in the initial stage and jump out of the local optimal solution compared with the other seven algorithms. From *f*_2_, the improved LFOA can reveal a fast convergence behavior and finally achieved the best solution. It is shown that the LFOA algorithm has the fastest convergence speed initially when using *f*_3_. It can be found that *f*_4_ and *f*_1_ have the same convergence phenomenon. From *f*_10_ and *f*_11_, the proposed LFOA shows a faster convergence rate in the early stages, but other algorithms are all trapped in local optima due to the weaker search capability. From *f*_12_, *f*_13_, the original FOA and the improved LFOA have a very fast convergence speed in the early stage, but the difference between FOA and LFOA is that FOA failed to escape from the local optimal solution in the later stage. From Figs. 2 and 3, we can conclude that the proposed algorithm not only has prominent advantages over other algorithms, but also converges very fast on most problems.

In summary, from Table 6 and Figs. 2 and 3, it can be seen that the improved LFOA has outstanding search advantages and faster optimization convergence than other counterparts.

### Results on the breast cancer diagnosis

In this section, the performance of the proposed model in the diagnosis of breast cancer has been thoroughly tested and analyzed. Table 7 shows the detailed results obtained by the LFOA-SVM model in the experiment. On average, the model achieves a classification accuracy of 93.83%, sensitivity of 91.22%, specificity of 96.53% and MCC of 0.8799.

**Table 7:** Classification performance of LFOA-SVM

Fold	ACC	Sensitivity	Specificity	MCC
#1	0.9362	0.9167	0.9565	0.8732
#2	0.8936	0.8148	1.0000	0.8074
#3	0.9362	0.8500	1.0000	0.8746
#4	0.9574	0.9565	0.9583	0.9149
#5	0.9574	1.0000	0.9167	0.9183
#6	0.9149	0.8400	1.0000	0.8431
#7	0.9787	1.0000	0.9565	0.9583
#8	0.9362	0.9000	0.9630	0.8694
#9	0.9362	0.9130	0.9583	0.8730
#10	0.9362	0.9310	0.9444	0.8672
Avg.	0.9383	0.9122	0.9653	0.8799
Std.	0.0234	0.0636	0.0272	0.0419

**Fig. 4:**
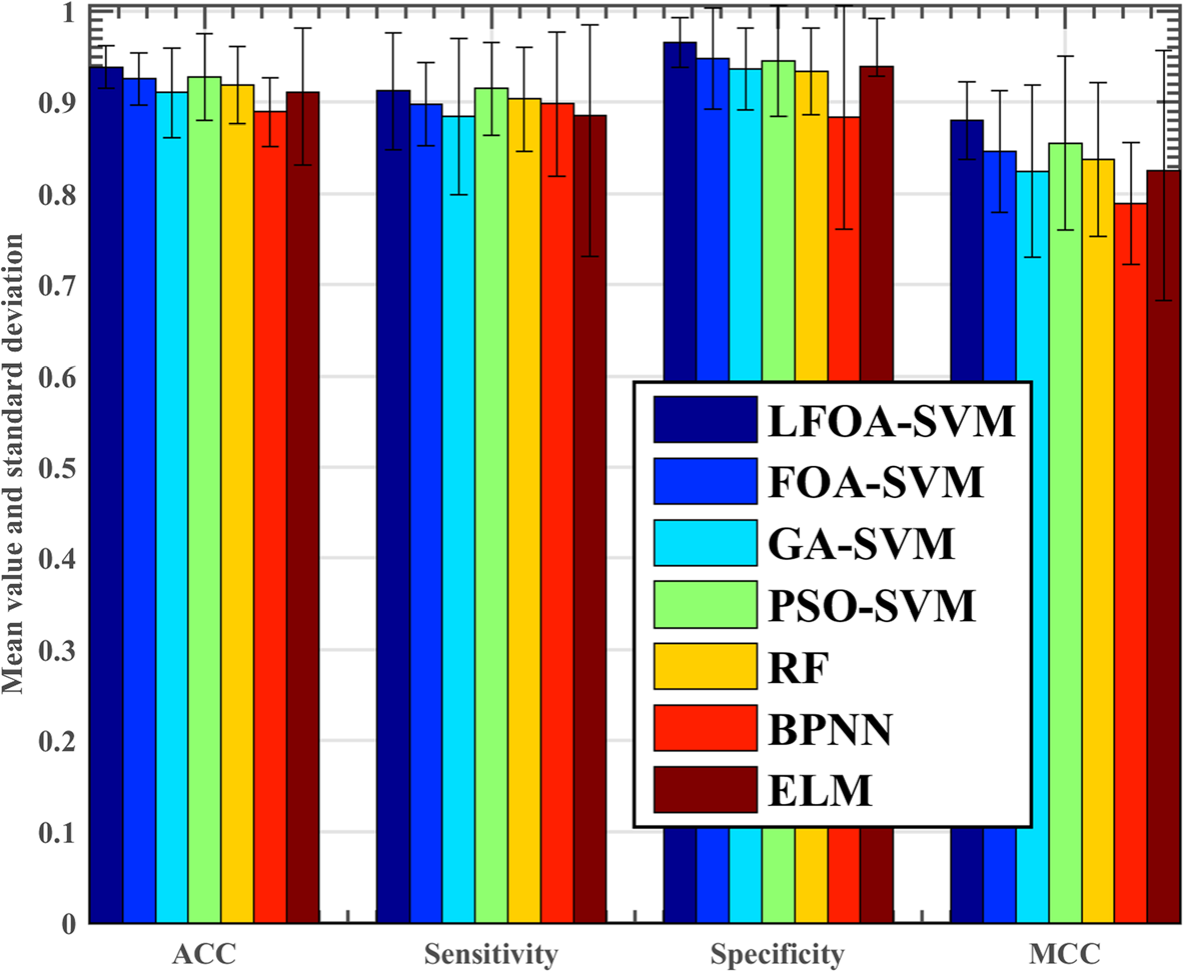
Classification performance obtained by the seven methods in terms of ACC, sensitivity, specificity and MCC

The proposed model and other six machine learning models including FOA-SVM, GA-SVM, PSO-SVM, RF, BP and ELM were tested simultaneously on the breast cancer dataset and the results are shown in Fig. 4. The figure reveals that the LFOA-SVM model is better than the FOA-SVM model in four evaluation metrics because compared with FOA-SVM, the ACC of LFOA-SVM is not only higher, but also the standard deviation is much smaller. On the ACC metric, the LFOA-SVM model obtained the best results. The results obtained by FOA-SVM and PSO model are very close behind the LFOA-SVM model, followed by RF, GA-SVM and ELM. The BP model has the worst result. On the Sensitivity metric, the PSO-SVM model obtains the best results. LFOA-SVM achieved the second place, followed by RF, BP, FOA-SVM and GA-SVM. The result obtained by ELM is the worst. On the Specificity metric, LFOA-SVM model obtained the best results. ELM achieved the second place. The results obtained by FOA-SVM and PSO model are very close behind the ELM, followed by GA-SVM and RF, the result obtained by GA -SVM and RF are very similar. The result obtained by BP is the worst. On the MCC metric, the LFOA-SVM model still obtains the best results. The PSO-SVM is in the next place, followed by FOA-SVM, RF, GA-SVM and ELM. The result obtained by BP is the worst.

For comparison purpose, we have also recoded the detailed results of the confusion matrix for LFOA-SVM and FOA-SVM. As shown in Table 8, we can see that LFOA-SVM correctly identifies 216 malignant tumors and 225 benign tumors, and misjudges 22 malignant tumors as benign tumors and 7 benign tumors as malignant tumors. FOA-SVM correctly identifies 215 malignant tumors and 220 benign tumors, misjudges 23 malignant tumors as benign tumors and 12 benign tumors as malignant tumors. The results indicate that LFOA is superior to FOA in the recognition of malignant tumors and benign tumors.

**Table 8:** Confusion matrix obtained by the proposed LFOA-SVM and FOA-SVM

No. of fold	LFOA-SVM		FOA-SVM	
1	20	3	27	2
	0	24	3	15
2	25	3	11	2
	1	18	3	31
3	26	3	25	2
	1	17	1	19
4	24	2	22	3
	1	20	2	20
5	16	3	24	2
	2	26	0	21
6	17	2	20	2
	1	27	0	25
7	25	0	22	1
	0	22	2	22
8	20	2	18	3
	0	25	0	26
9	22	2	18	4
	0	23	1	24
10	21	2	28	2
	1	23	0	17
Sum	216	22	215	23
	7	225	12	220

**Fig. 5:**
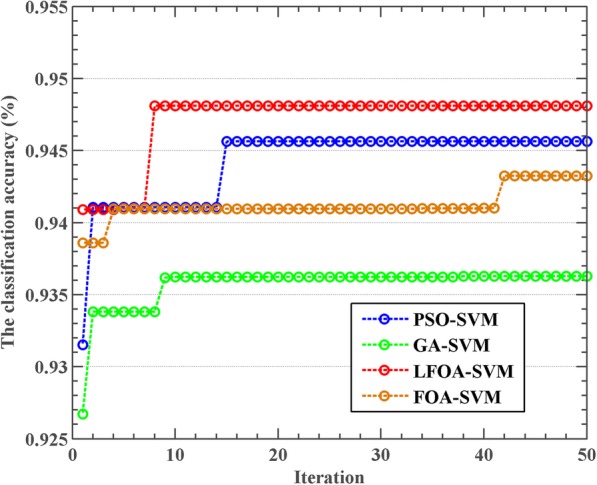
Relationship between the iteration and training accuracy of LFOA-SVM, FOA-SVM, PSO-SVM, and GA-SVM

In order to comprehensively evaluate the performance of the model, the convergence curve of the model based on the meta-heuristic algorithms in the training process is also compared and analyzed. The convergence curves of the four models are presented in Fig. 5. As shown, LFOA-SVM model not only has a very fast convergence speed but also achieves the highest classification accuracy. However, FOA-SVM model has a slow convergence speed. The main reason is that LF mechanism can improve the global search ability of FOA. Inspecting the curves in Fig. 5, The FOA-SVM model needs more iterations to converge and the obtained solution is not better than that of LFOA-SVM model. The GA-SVM model converges after a few iterations, which reveals the GA has a weak global search capability, it takes a long time to jump out of the local optimum, and the final result is not satisfactory.

## Discussions

In this study, a new support vector machine model (LFOA-SVM) based on LF strategy enhanced FOA is proposed to diagnose the breast cancer. The main novelty lies in the improved FOA strategy (LFOA) was proposed for the first time and applied to predicting the breast cancer from the perspective of the high-level features as well. Compared with the original FOA and other optimizers, LFOA can achieve the better solution and has a faster convergence speed as well. LFOA has aided SVM to achieve much more suitable parameters for learning and thus get the higher prediction performance for breast cancer diagnosis. The experimental results have demonstrated that the LFOA-SVM model has achieved better performance than the other competitive counterparts.

The main contributions of this study are as follows:

- First, in order to fully explore the potential of the SVM classifier, we introduce a levy flight strategy-enhanced FOA to adaptively determine the two key parameters of SVM, which aided the SVM classifier in more efficiently achieving the maximum classification performance.
- The resulting model, LFOA-SVM, is applied to serve as a computer-aided decision-making tool for diagnosing the breast cancer from high-level features for the first time.
- The proposed LFOA-SVM method achieves superior results and offers more stable and robust results when compared to the other SVM models.

## Conclusions

This paper has developed an effective LFOA-SVM method which can well diagnose the breast cancer in clinical diagnosis and provide doctors with meaningful clinical decision. The proposed method has achieved a classification accuracy of 93.83%, sensitivity of 91.22%, specificity of 96.53% and MCC of 0.8799 for breast cancer diagnosis based on the high-level features.

Improving the LFOA method via introducing the mechanisms such as mutation strategy or the opposition-based learning strategy is our future research direction. In addition, we will plan to apply the method to other related disease diagnosis problems.

## Abbreviations

ACC: Classification accuracy
Avg: Average
BA: Bat algorithm
BPNN: Back propagation neural network
CV: Cross validation
DA: Dragon fly algorithm
ELM: Extreme learning machine
FOA: Fruit fly optimization algorithm
FOA-SVM: SVM model based on original FOA
FPA: Flower pollination algorithm
LF: Levy flight
LFOA: FOA enhanced by LF strategy
LFOA-SVM: SVM model based on original FOA
MCC: Mathews correlation coefficient
MFO: Moth-flame optimization
PSO-SVM: SVM model based on original PSO
RF: Random forest
SCA: Sine cosine algorithm
Std: Standard deviation
SVM: Support vector machine
